# Challenges and Choices Amidst Military Conflict: The Present and Future of Medical Education and Healthcare in Sudan

**DOI:** 10.1002/hsr2.70334

**Published:** 2025-01-08

**Authors:** Sohaib Mohammed Mokhtar Ahmed, Mohanned Bushra Masaad, Mohamed Salah Abdulrazeg Abdulrahman, Elnazeir Mohamed Ibrahim Mohamedzain

**Affiliations:** ^1^ Faculty of Medicine and Health Sciences University of Gadarif Gadarif Sudan; ^2^ Faculty of Medicine Alzaiem Alazhari University Khartoum Sudan

**Keywords:** e migration, healthcare, medical education, medical students, Sudan

## Abstract

**Background:**

The situation for medical education and healthcare in Sudan has been challenging for the recent years, and emigration of physicians is an ongoing problem threatening the healthcare system. We conducted this study to understand the future plans of medical students and their perceptions regarding their medical education and healthcare system.

**Method:**

We performed this cross‐sectional study at five public and private Sudanese medical schools in November 2023. We distributed the questionnaire electronically through an Online Google Form via social media platforms, such as WhatsApp Messenger. Descriptive statistics were employed for the data analyses.

**Results:**

The mean age (±SD) of the students was 22.2 (±2.7) years, and females constituted 61.8% (397) of the study sample. About 18% of the students rated their medical training as excellent, and half of them (50%) said that it was good. Nearly half of the students believed the quality of healthcare services in hospitals was poor (46%) along with a shortage or severe shortage in its number (49%). A majority (58.4%) thought frequently or constantly about leaving Sudan after graduation. Reasons given for leaving included better personal lifestyle, avoiding conflict, advanced training, and better professional opportunities and salary. Leading motives for staying included the pull of friends and family and a sense of responsibility to the country.

**Conclusion:**

This study indicated the pressing need for comprehensive interventions to address the challenges faced by medical students in Sudan. The strong desire to emigrate increased by the current conflict, coupled with concerns about the healthcare system and medical education, poses a significant threat to the future of healthcare services in the region.

## 1 Introduction

Conflict or violent insecurity demonstrate a persistent international problem. By 2030, nearly half of the world's poorest people are expected to live in countries affected by fragility, conflict, and violence [1]. Sudan is suffering from a profound humanitarian crisis following the start of military conflict on 15th April of 2023 between the Sudanese Armed Forces and the Rapid Support Forces in the capital city of Khartoum, North Kordofan, Darfur, and River Nile states. The existence of conflict or insecurity within or between states affects access to regular civil activities such as employment, healthcare, and education [2, 3]. Armed conflict has a deep influence on health services. Health facilities are impaired, medicines and supplies are insufficient, communications and transport are interrupted, and the loss of professional staff damages health services significantly. Conflict has motivated medical staff to pursue employment outside of their home countries, as seen in the cases of Cuba, Lebanon, and Liberia [4, 5, 6].

The recent war conflicts have had a profound impact on medical education, as highlighted by several studies. Hodkinson et al. provide valuable insights into the challenges encountered by medical students in Ukraine during the COVID‐19 pandemic and the Russian military invasion. The disruptions caused by these events resulted in a shift to remote learning, leading to a significant gap in clinical education [7]. To address this gap, the University of Cambridge organized an intensive clinical elective program for Ukrainian medical students. Another study focused on the effects of the ongoing war in Ukraine on the quality of medical education. The research, conducted among foreign students studying in Ukraine, reveals a decline in satisfaction with the quality of education during the conflict. Limited practical training opportunities, restricted access to clinical facilities and patients, and decreased safety and comfort in the learning environment contribute to this decline [8]. Overall, these studies, along with the research conducted by Mayer et al., demonstrate that war conflicts significantly disrupt medical education [9]. The restrictions, changes in teaching and learning, increased workloads, and financial constraints pose substantial challenges to students, faculty, staff, and the healthcare system. Delays in studies, missed learning objectives, and threats to the quality of healthcare education are common consequences [9].

Medical education started in Sudan in 1924 with the establishment of the Gordon School of Medicine, now known as the Faculty of Medicine at the University of Khartoum. In the late 1970s, two other public medical schools were established, resulting in a total of three medical schools in the country. With an annual intake of 250 students, these schools provided a well‐balanced education within the available resources, ensuring that graduates were well‐trained and learned at a high standard [10]. However, in 1991, the government initiated the “Revolution in Higher Education,” which led to the opening of new public and private medical schools. As a result, the number of medical schools in Sudan increased to 72, and the annual intake of students surpassed 5000 [11, 12]. Unfortunately, this rapid expansion was not accompanied by a corresponding increase in training facilities and well‐trained mentors. Consequently, the large number of students being taught often exceeds the available resources and affects the amount of hands‐on training they receive [11, 12, 13].

Physicians' emigration in Sudan has been a significant concern. Since the early 1960s, the country has lost nearly 60% of its physicians to emigration. In 2003, it was estimated that around 12,000 out of 21,000 medical graduates registered in the Sudanese Medical Council were working abroad [14]. Currently, around 3426 highly trained Sudanese physicians with postgraduate experiences in various medical disciplines are employed and living abroad [15]. Moreover, the trend of physicians leaving Sudan continues, with recent estimates showing that 30% of the 3000 yearly medical graduates emigrate annually. While male physicians constitute the majority of migrants, there has been an observed increase in female medical emigration [16]. The main causes of medical emigration are mainly financial, together with a shortage of advanced training and profession development opportunities, both of which are considered important for physicians in Sudan [17]. However, no studies have investigated the influence of the current military conflict on physicians' emigration.

With the retirement and emigration of many physicians in Sudan, concerns have been elevated about the future delivery of medical care for the country's 48 million people. Targeting medical students in this study is justified as they represent a critical juncture where early interventions can potentially reduce the likelihood of their emigration. By identifying their future career plans, perceptions of education quality, and concerns about the healthcare system, we can develop targeted strategies to address these factors and create a supportive environment that encourages them to stay and contribute to the healthcare workforce in Sudan. Understanding and addressing these issues at an early stage can help mitigate the factors that drive medical student emigration and ultimately improve the retention of skilled professionals within the country.

### 1.1 Study Objectives

The study aimed to comprehensively understand the career aspirations, future plans, motivations for staying or leaving Sudan, perceptions about medical training, and healthcare services among medical students in East Sudan.

## 2 Method

### 2.1 Study Design

This cross‐sectional study was conducted among medical students at five medical schools in East Sudan in November 2023. The findings and methodology of this study were meticulously reported in the manuscript in accordance with the Strengthening the Reporting of Observational Studies in Epidemiology guidelines.

### 2.2 Study Population and Setting

The study included all medical students aged at least 18 years old who agreed to participate from five universities in East Sudan: Red Sea University, Kassala University, Gadarif University, Gezira University, and Al‐Butana University. Dental, pharmacy, nursing, and medical laboratory students were excluded.

### 2.3 Sampling

We estimated the sample size using the Cochrane formula, given the unavailability of official population records of medical students in Sudan. Assuming a population proportion of 50%, a margin of error of 5%, and a confidence interval of 95%, the minimum sample size required was calculated to be 385 participants. Ultimately, 642 medical students participated in the study.

Convenience sampling was employed due to the challenges in accessing official student records. An online survey using Google Forms was distributed among medical students via popular social media platforms including WhatsApp, Telegram, and Facebook. These platforms are widely utilized by medical students in Sudan, especially during online studies in Sudan during the ongoing war. The survey was anonymous and took approximately 8 min to complete and there was no incentive provided for filling in the survey.

### 2.4 Study Instrument

We utilized a previously validated questionnaire from similar studies conducted in Afghanistan and Iraq [18, 19]. The questionnaire consists of four parts. The first section includes questions about participants' socio‐demographic characteristics (10 questions). The second section asked the students about their future career plans and the effects of conflict and insecurity on their professional choices (19 questions). The third section inquires about the quality of their current medical education (10 questions) utilizing Likert‐scale questions (strongly agree, agree, not sure, disagree, and strongly disagree). Finally, the last section focuses on their perceptions of healthcare in Sudan (12 questions) with choices of Excellent, Good, Fair, and Poor.

### 2.5 Data Management

To ensure robust data collection and maintain data integrity, we implemented logic and validation checks in the online survey instrument. These checks included mandatory response fields, range restrictions, and consistency checks for selected responses. These measures aimed to minimize errors and ensure the reliability of the collected data. Data analysis was conducted using Jamovi version 2.3.21.0 [20]. Categorical data were described using frequencies and percentages, while numerical data were presented as medians and interquartile ranges. Socio‐demographic factors were compared with students' responses using the chi‐square test or Fisher's exact test, as appropriate. A *p* value of 0.05 or less was considered statistically significant.

## 3 Results

### 3.1 Demographic Features

The study included 642 medical students from five institutions in East Sudan. Most participants were female (61.8%), with a mean age of 22.2 years. Regarding academic performance, students were categorized into three groups based on their GPA: the lower third, middle third, and higher third. Approximately 35.2% of students placed themselves in the higher third of their class, while 60.6% were in the middle third. The majority (84.0%) reported having moderate socioeconomic status (Table 1).

**Table 1:** Socio‐demographic features.

	*N* (%)
**Age**	
Mean (SD)	22.2 (2.7)
**Sex**	
Female	397 (61.8%)
Male	245 (38.2%)
**University type**	
Government University	459 (71.5%)
Private University	183 (28.5%)
**Study year**	
Clinical years [4th, 5th, 6th years]	250 (38.9%)
Preclinical years [1th, 2th and 3th years]	392 (61.1%)
**Academic ranking**	
Lower third	27 (4.2%)
Middle third	389 (60.6%)
Higher third	226 (35.2%)
**Economic status**	
Low	65 (10.1%)
Moderate	539 (84.0%)
high	38 (5.9%)
**Presence doctor in family or close relatives**	
No	183 (28.5%)
Yes	459 (71.5%)

### 3.2 Future Professional Career Plans

**Figure 1:**
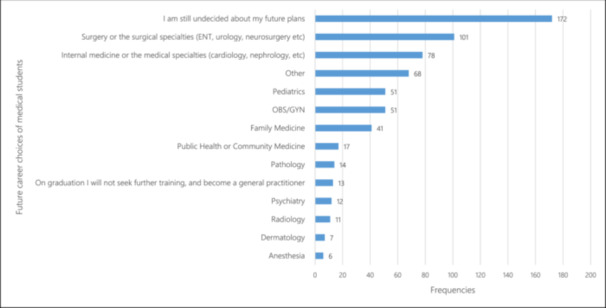
Future career choices of medical students.

**Figure 2:**
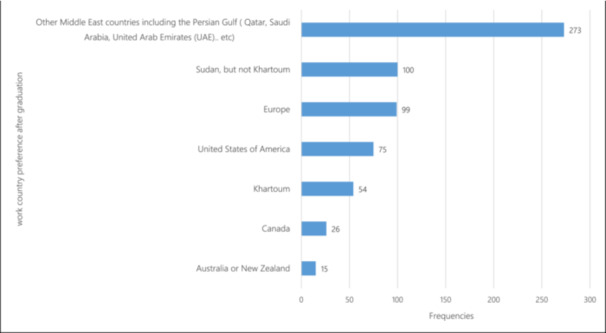
future plans of medical students regarding their postgraduation training.

**Figure 3:**
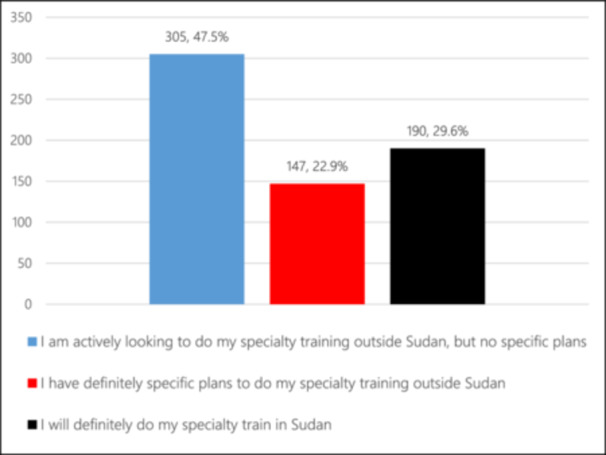
Medical students work country preference after graduation.

Around one‐quarter of the students (26.8%) were undecided about their future career plans. Popular career choices included surgical specialties (15.7%) and internal medicine (12.1%) (Figure 1). Approximately 47.5% expressed a desire to pursue specialty training abroad (Figure 2), with Middle Eastern countries being the preferred destination for 42.5% of students (Figure 3). Notably, 58.4% reported frequent or constant thoughts about leaving Sudan due to career and lifestyle opportunities abroad.

### 3.3 Motivation for Staying or Leaving Sudan

The most commonly cited reason for staying in Sudan was the desire to remain close to family and friends (55%). Conversely, motivations for leaving were primarily driven by the pursuit of a better lifestyle (72%) and avoiding conflict (73%). Additionally, advanced training and better professional opportunities were significant factors encouraging emigration (Table 2).

**Table 2:** Medical student's motivations for leaving or staying in Sudan.

	Most important	Somewhat important	Not very important	Least important
Motivation for Staying in Sudan:				
To be with family and friends.	353 (55%)	191 (30%)	70 (11%)	28 (4.4%)
Familiar with health care system in Sudan.	269 (42%)	214 (33%)	97 (15%)	62 (9.7%)
Responsibility to help my country.	335 (52%)	178 (28%)	71 (11%)	58 (9.0%)
Positions I can get here are better than I can get outside of Sudan.	168 (26%)	175 (27%)	159 (25%)	140 (22%)
Personal life style in Sudan is what I like.	213 (33%)	160 (25%)	135 (21%)	134 (21%)
Motivation for Leaving Sudan:				
Better personal lifestyle.	463 (72%)	113 (18%)	42 (6.5%)	24 (3.7%)
Avoid war and conflict.	470 (73%)	125 (19%)	35 (5.5%)	12 (1.9%)
Looking for advanced training.	517 (81%)	94 (15%)	20 (3.1%)	11 (1.7%)
Better professional opportunities.	502 (78%)	106 (17%)	23 (3.6%)	11 (1.7%)
Better salary and working conditions.	483 (75%)	124 (19%)	23 (3.6%)	12 (1.9%)

### 3.4 Perceptions about Medical Training

Over half of the respondents (56%) agreed that their clinical rotations were well organized. However, 42% expressed concerns about the interference of faculty members' private practice responsibilities with their teaching roles. Despite these concerns, 68% of students rated the overall quality of their medical training as good or excellent. Nearly half of the students believed that the quality of medical education in Sudan was worse compared to other countries in the region (Table 3).

**Table 3:** Medical student's perceptions about their medical education.

	Strongly agree	Agree	Not sure	Disagree	Strongly disagree
Clinical rotations are well organized to help me learn the topics.	121 (19%)	237 (37%)	155 (24%)	57 (8.9%)	72 (11%)
Access to current textbooks and journals is generally good.	79 (12%)	270 (42%)	131 (20%)	91 (14%)	71 (11%)
Faculty conduct lectures when scheduled.	39 (6.1%)	202 (31%)	176 (27%)	125 (19%)	100 (16%)
Faculty keep up to date in the latest developments in their field.	62 (9.7%)	219 (34%)	203 (32%)	89 (14%)	69 (11%)
Faculty are generally good teachers with a strong interest in helping us learn	122 (19%)	248 (39%)	125 (19%)	84 (13%)	63 (9.8%)
Faculty private practice responsibilities interferes with teach.	50 (7.8%)	214 (33%)	218 (34%)	106 (17%)	54 (8.4%)
The basic science curricula were well organized to help me learn.	79 (12%)	296 (46%)	131 (20%)	76 (12%)	60 (9.3%)
Faculty knowledge and dedication to teaching is very high.	67 (10%)	234 (36%)	174 (27%)	102 (16%)	65 (10%)
		Excellent	Good	Fair	Poor
Overall perceived quality of medical training at your Medical School.		117 (18%)	322 (50%)	119 (18%)	84 (13%)

### 3.5 Perception of the Healthcare System in Sudan

When evaluating the healthcare services in Sudan, 46% of participants rated hospital care as poor, and only 30% considered it to be good or excellent. Private clinics were viewed more favorably, with 20% rating them as excellent and 42% as good. The availability of laboratory tests and medicines received poor ratings from 38% and 47% of respondents, respectively. Salaries for doctors were also a major concern, with only 25% of students rating them as good or excellent, while 53% considered them poor. Approximately 48% of participants deemed the working conditions for doctors to be poor, citing safety and security issues as additional concerns (Tables 4 and 5).

**Table 4:** Medical student's perceptions about health care system in Sudan.

	Excellent	Good	Fair	Poor	Don't know
Quality of health care received at private clinics.	128 (20%)	272 (42%)	122 (19%)	77 (12%)	43 (6.7%)
Quality of health care received at hospitals.	45 (7.0%)	149 (23%)	128 (20%)	293 (46%)	27 (4.2%)
Attitude and concern of doctors for patients.	77 (12%)	249 (39%)	138 (21%)	129 (20%)	49 (7.6%)
Salary and Income for doctors.	51 (7.9%)	112 (17%)	81 (13%)	342 (53%)	56 (8.7%)
Availability of laboratory tests when needed.	39 (6.1%)	180 (28%)	142 (22%)	243 (38%)	38 (5.9%)
Attitudes and concern of other health care workers for patients.	38 (5.9%)	186 (29%)	167 (26%)	187 (29%)	64 (10.0%)
Quality of health care received at primary health care clinics.	37 (5.8%)	166 (26%)	175 (27%)	207 (32%)	57 (8.9%)
Availability of medicines and supplies when needed.	40 (6.2%)	117 (18%)	142 (22%)	302 (47%)	41 (6.4%)
Attitude of patients and families toward doctors.	56 (8.7%)	195 (30%)	157 (24%)	180 (28%)	54 (8.4%)
Safety and security of doctors.	52 (8.1%)	167 (26%)	106 (17%)	271 (42%)	46 (7.2%)
Working Conditions for doctors.	41 (6.4%)	143 (22%)	96 (15%)	308 (48%)	54 (8.4%)

**Table 5:** Medical student's perceptions about the availability of health care services.

	More than enough	Adequate	Shortage	Severe shortage
The number of hospitals.	105 (16%)	220 (34%)	252 (39%)	65 (10%)
The number of Primary Health Care Clinics (PHCCs).	69 (11%)	272 (42%)	252 (39%)	49 (7.6%)
The number of private clinics.	141 (22%)	296 (46%)	157 (24%)	48 (7.5%)
The number of nurses in hospitals and clinics.	100 (16%)	297 (46%)	201 (31%)	44 (6.9%)
The number of technicians (laboratory, x‐ray, pharmacy etc.).	74 (12%)	239 (37%)	257 (40%)	72 (11%)
The availability of medicines and supplies when needed.	77 (12%)	162 (25%)	288 (45%)	115 (18%)
The availability of laboratory tests when needed.	91 (14%)	196 (31%)	264 (41%)	91 (14%)

### 3.6 Association Analysis

Gender significantly influenced perceptions of practice attraction over the past 3 years (*χ*² = 31.3, *p* < 0.001), with males more likely to report improvement. University type also showed notable differences, with government university students perceiving better faculty relationships (*χ*² = 4.70, *p* = 0.030) and a stronger attraction to medical practice (*χ*² = 13.3, *p* = 0.004). Study year significantly impacted specialty training plans, as clinical‐year students were more likely to plan training abroad (*χ*² = 6.00, *p* = 0.050), and those in preclinical years had stronger connections with faculty (*χ*² = 15.1, *p* < 0.001).

## 4 Discussion

The goal of this study was to explore the future career plans of medical students in Sudan, as well as their perceptions of the quality of education and healthcare system in Sudan. The aspiration to leave Sudan after graduation, for both professional and personal reasons, was found to be very strong, with the ongoing armed conflict playing a significant role. The overall perception of medical education was largely positive, although there were significant concerns regarding faculty working conditions and emigrations. In terms of the healthcare system, rising concerns were identified regarding the availability of hospitals, medical supplies (such as medications, laboratory tests, and equipment), and the quality of healthcare services provided. Medical students also expressed major concerns about the working conditions, salaries, safety, and security of physicians.

The study revealed a strong desire among medical students to emigrate or pursue their training outside Sudan. This trend was also observed in other conflict‐affected countries, such as Iraq and Afghanistan [18, 19], suggesting the potential impact of ongoing conflicts on medical students' career choices as half of the students in our study reported that their career choices and future plans were highly influenced by the ongoing conflict. The considerable outflow of physicians from Sudan in recent years has negatively affected the amount and quality of healthcare services in the country. Shortages of physicians have been reported in many states [21], further exacerbating concerns about the future of healthcare services. The motivation for students to leave the country in our study was driven by the desire for a better personal lifestyle, to avoid war and conflict, pursue advanced training, and seek better professional opportunities and salaries. Interestingly, these same factors were identified as leading causes of health professionals' emigration from Sudan 12 years ago by Ibrahim et al. [22] and 18 years ago by Badr et al. [14]. Moreover, the negative perception of practicing medicine as a young doctor in Sudan may contribute to the emigration problem, which can be attributed to poor working conditions, salaries, and doctors' security, as stated by the students themselves. These challenges may be further exacerbated by the ongoing conflict.

Previous studies revealed that enrollment in postgraduate training programs affect students' choices regarding permanent residency in their home country [23, 24]. Internal medicine and surgery were commonly chosen career paths among medical students in our study. The popularity of these specialties may be attributed to their advanced and high‐level training requirements, which are typically not available in Sudan. Approximately half of the students reported that medical training in Sudan was worse or much worse than training in other regions. These findings highlight the urgent need for effective measures to address this issue.

The overall perception of medical students regarding their medical training was generally positive, with 68% of students rating the overall quality of their training as good or excellent. Although one might argue that students don't have a benchmark for making this comparison, they are still able to evaluate how effectively their training equips them for medical practice in Sudan. Students expressed positive perceptions of their faculty member, describing them as good and highly dedicated teachers. However, there was a growing concern among students regarding the interference of faculty members' private practices in their teaching responsibilities, which may be connected to the negative perception that faculty member do not conduct lectures as scheduled. The economic challenges and complex situation in Sudan in recent years, coupled with the low salaries of faculty members, have put significant economic pressure on the facilities, contributing to students' concerns. These circumstances may also lead to faculty emigration outside Sudan, as 68.7% of students reported knowing one or more faculty members who had emigrated, highlighting the scope of the problem. Medical education in Sudan has had positive influences on the health system, both locally and regionally [11], making it crucial to maintain its quality by improving faculty situations and preventing the negative impact of emigration or conflicts of interest.

Regarding the healthcare system in Sudan, students generally held negative perceptions and expressed multiple concerns. Half of the students reported a shortage or severe shortage of hospitals and primary healthcare clinics. Furthermore, the available hospitals were perceived to have poor healthcare quality, including deficiencies in medicine supply, technicians, nurses, pharmacies, and necessary laboratory tests. Despite the growing production of healthcare professionals each year to meet the system's needs, there is still a shortage in human resources [25]. The main challenges include the emigration of trained professionals, similar to many African countries, as well as poor managing and inadequate dissemination of present physicians and paramedics [26]. Sudan has lost nearly 60% of its physicians due to emigration [27]. In addition to exterior threats such as brain drain, continued economic sanctions, and departure from South Sudan, which have affected economic support and hindered goal achievement, internal weaknesses within the healthcare system also persist [28]. Poor logistical supply of equipment, disposables, and drugs has been observed as well [28].

Physician working conditions were a major concern, with approximately half of the students describing them as poor, including issues related to safety, security, and salaries. A study conducted in Khartoum by Ali et al. indicated that half of OB/GYN physicians experienced workplace violence (WPV) in the form of verbal, physical, or sexual incidents [29]. Furthermore, Elhadi et al. reported that during the COVID‐19 pandemic, 78.3% of health workers in Sudan experienced violence, with 65.8% facing it more than three times [30]. WPV in healthcare settings has detrimental effects on healthcare workers, leading to increased absences from work, decreased performance and job satisfaction, and poor mental health, all of which contribute to suboptimal patient care [31]. Additionally, suboptimal patient care and a lack of high‐performing healthcare employees create a vicious circle that perpetuates WPV [32]. Moreover, health worker salaries in Sudan are low compared to North Africa and even poorer countries in sub‐Saharan Africa [33]. At the period of this study, junior physicians in Sudan made an average of 100,000 SDG (US$ 170) per month. Considering the existing economic crisis in Sudan, it is unlikely that policymakers can implement good salary increases to preserve physicians, making this aspect difficult to address through policy reform [33].

It is important to recognize that the socioeconomic status of the respondents, with the majority being in the moderate to high‐income category (84.0%), may have influenced their perspectives and responses in the study. Higher socioeconomic status could be associated with better access to resources such as private healthcare, advanced educational tools, and broader opportunities for international exposure. These factors may explain why a significant portion of students expressed dissatisfaction with the local healthcare and educational infrastructure, and a strong desire to emigrate for better professional and personal prospects. Their financial stability might also have provided them with greater flexibility in considering opportunities abroad, as they are less dependent on the local system for their immediate economic survival. Future studies should aim to include a broader range of socioeconomic backgrounds to fully capture how different financial circumstances might shape medical students' perceptions and career choices.

### 4.1 Limitations

There are some limitations to consider. The study was restricted to East Sudan due to the current complex situations in other parts of the country and issues with internet instability, which may limit the population representation. The online nature of the questionnaire may have affected its distribution due to electricity and internet instability during the war. Also, the population size was not available; therefore, we couldn't estimate the response rate. Furthermore, the study relied on self‐reported data, which may be subject to recall bias or social desirability bias, in addition to selective bias. Additionally, the cross‐sectional design of the study limits our ability to establish causal relationships or assess changes over time. Our study primarily relied on a structured survey instrument that queried participants about specific factors related to students' future professional plans. While this approach allowed us to systematically gather data on predetermined variables, it indeed limited the opportunity for participants to spontaneously mention additional factors not included in the survey. Lastly, the study did not explore the perspectives of key stakeholders such as faculty members, healthcare administrators, or policymakers, which could have provided a more comprehensive understanding of the issues at hand. Future research should address these limitations to further inform interventions and policies aimed at improving the healthcare system for medical students and professionals.

### 4.2 Implications

Based on the findings of this study, urgent and effective actions are necessary to tackle the concerning trends observed among medical students in Sudan. The strong desire to leave the country after graduation, coupled with negative perceptions of the healthcare system and, pose significant challenges to the future of healthcare services in the region. Prioritizing efforts to retain and attract medical professionals by improving working conditions, ensuring competitive salaries, and enhancing safety and security measures is crucial. Additionally, addressing the shortage of hospitals, medical supplies, and healthcare facilities, as well as strengthening the quality of medical training, should be key areas of focus. Furthermore, efforts should be made to prevent faculty emigrations and conflicts of interest, as they can have a detrimental impact on the quality of education and healthcare delivery. By taking proactive measures to address these issues, Sudan can work towards creating a more favorable environment for medical students, healthcare professionals, and ultimately, the overall healthcare system.

## 5 Conclusions

In conclusion, the findings of this study highlight the pressing need for comprehensive interventions to address the challenges faced by medical students in East Sudan. The strong desire to emigrate increased by the ongoing conflict, coupled with concerns about the healthcare system and medical education, poses a significant threat to the future of healthcare services in the region. Efforts should be directed towards improving working conditions, ensuring competitive salaries, and enhancing safety and security measures for medical professionals. Additionally, addressing the shortage of healthcare facilities, strengthening the quality of medical training, and preventing facultye emigrations are crucial steps. By implementing these measures, Sudan can strive towards a more sustainable and robust healthcare system.

## Author Contributions

Sohaib M.M. Ahmed conceived the study, created the data instruments, analyzed the data, and interpreted the results and prepared the final paper. Mohanned B. Masaad participated in the study design and creation of the instruments, collection of the data, and manuscript drafting. Mohamed S.A. Abdulrahman participated in the review of the instruments, interpretation of the results as well as manuscript preparation. All listed authors were tangled in review and interpretation of the data and final approval of the manuscript.

## Ethics Statement

Ethical approval was obtained from Gadarif University's Medical Research Ethics Committee (Ref. No.: GU/FM/REC/Q3.10.23.4). All participants provided informed consent, and the study was conducted in accordance with the principles outlined in the Declaration of Helsinki.

## Conflicts of Interest

The authors declare no conflicts of interest regarding this study or its publication.

## Transparency Statement

The lead author Sohaib Mohammed Mokhtar Ahmed affirms that this manuscript is an honest, accurate, and transparent account of the study being reported; that no important aspects of the study have been omitted; and that any discrepancies from the study as planned (and, if relevant, registered) have been explained.

## Data Availability

Data used in this study are available upon reasonable request from the corresponding.
